# Sports and Social Interaction: Sports Experiences and Attitudes of the Urban Running Community

**DOI:** 10.3390/ijerph192114412

**Published:** 2022-11-03

**Authors:** Jia Yang, Fang-Yuan Ju, Zu-Guo Tian

**Affiliations:** 1 College of Physical Education, Hunan University, Changsha 410082, China; 2 College of Physical Education, Yangzhou University, Yangzhou 225012, China

**Keywords:** marathon, running group, runners, social interaction, qualitative research

## Abstract

Social change and development influence the motivational factors of people’s sports participation, exposing the need for socialization and interaction. The purpose of this study is to investigate the interaction pattern of the urban running community and the behavior pattern of runners with the help of social interaction theory, and to explore the inner connection between their community movement and social interaction. Ten senior members of marathon urban running societies were selected to conduct two rounds of in-depth interviews which were analyzed using qualitative thematic analysis to understand the sports participation experiences and social interactions of running society members. The study found that the whole interaction process of running groups is accomplished through three aspects: examination of self, adjustment with reference to others, and a sense of group belonging as the core consciousness. On the other hand, the social attributes of running groups can advance the personalization process of runners in society, which is mainly characterized by self-presentation and external constraints, self-requirement and group constraints, intergroup interaction and group identity reorganization. In addition, the unique community culture, standardized group organization and harmonious activity atmosphere will stimulate runners’ interest in running, strengthen community communication and establish stable community relations, etc., which in turn will bring about continuous interactive behaviors.

## 1. Introduction

China is at the historical moment of moving from a large sports country to a strong sports country [1]. Plus, forming a working pattern of “government-led, socially coordinated, public participation, and guaranteed by the legal system” for national fitness [2]. People’s economic conditions have gradually improved under social transformation. People have sufficient leisure time, more diversified sport needs, and gradually increasing awareness of sports participation [3]. Boosted by urban development, the “marathon” boom has come, and the number of participants has continued to increase, with marathon runners covering 31 provinces in China with 7.12 million participants, an increase of 2.5 times compared to 2016. With the policy trend, economic globalization and regional integration, China’s urban form has shifted from “labor” to “leisure”, and the social structure and form have influenced the changes in people’s lives and values [4,5]. Social changes in China have been influencing the forms of sports participation, but also revealing social and emotional needs [6]. Gau (2022) states that leisure sport activities diversity (LSD) can be used to assess a country’s development indicators, reflecting people’s well-being and satisfaction [7]. As marathon sport entered a golden period of rapid development after 2015, the factors affecting the expansion of marathon space in China are changing and correlated [8]. Group running has also become a popular social phenomenon in China [9]. Marathon running groups have emerged in Chinese cities, formed by runners on their own initiative, with a large number of participants. Running groups have a positive impact on people’s sports participation. Studies have shown that running can improve chronic diseases, promote cardiovascular health and reduce the risk of death [10,11], and that running is also helpful for mental health, which can have a placebo effect, especially participating in community-based running, which can improve well-being and relieve mental stress [12,13]. Members of running groups can experience a pleasant and relaxing vibe, escaping from daily liabilities and fatigue, even for a short while; they can also give full attention to the opportunity to make new friends and network [14]. Through running groups, runners can share running news and personal insights, exchange running techniques and routes, organize running group events, and forward competition information in an online or offline manner to achieve social interaction and satisfy their social demands. In this way, peer interaction exercise positively influences participants’ motivation and confidence [15]. This study takes the members of marathon urban running groups as the objects of investigation, explores the phenomenon of sports participation in urban running associations with the help of social interaction theory, and studies the sports participation patterns of marathon association runners to understand their sports motivation and participation attitudes.

## 2. Literature Review

In social life, we are constantly carrying out complex activities, which means that multiple social interactions are also constantly taking place. Current research has focused on social interaction and capital [16], social interaction and geospatial relationships [17], and social network relationships [18,19], among others. Social interactions are influenced by characteristics of the residential environment as well as by personal and mobility attributes. Social networks may lead to heterogeneity of interactions, but interacting groups formed by geographical relations remain the basis of social interactions [20].

Physical activity is also a beneficial way to promote social interaction. Several studies have shown that marathons can lead to deep levels of sports participation and social interaction [21,22,23]. Marathons promote social network formation and stimulate sports consumption through social interaction in a way that creates intergenerational influence, peer influence, and media influence [24,25]. During a marathon, runners are able to experience strong emotions and have the opportunity to build social relationships [26]. The stronger the psychological relationship of running behavior, the more prominent the frequency and depth of their athletic engagement and interaction [27]. Rupprecht and Matkin (2012) explored the meaning of women’s participation in multiple marathons through in-depth interviews [28]. The results suggest there are five motifs for women’s participation, that is, emotion, pride, intimacy, inspiration, and transformation [28]. Compared to women, men are primarily motivated by a tendency to increase speed, reach their potential (i.e., “personal goal achievers”) and compete with others (i.e., “competitive achievers”) [29]. Sports participation can be a channel to develop oneself and have himself or herself integrated into society. On the other hand, runners’ participation strategies and effects also closely relate to their role orientation; for novice runners, via intra-group and intergroup interactions, they can progressively identify with the social values and behavioral patterns shared by other marathoners, as a result forming a continuous motivation to participate [21]. A marathon running group is a self-organized, self-managed, and self-developed group of marathon enthusiasts, in which multiple interactive relationships are established. Taiwanese scholars have conducted a series of studies on the reasons, history, and effective participation of marathon group. They maintained that participating in marathon events may be conducive to developing a community, establishing an important interpersonal network for individuals, and even constituting a critical part of personal sense of self; that is, such event can promote an individual’s sense of identity [15]. However, the demands vary by location, with urban residents having an intense demand for marathon participation and a 15% higher motivation to participate compared to rural residents [26]; moreover, family members and family structure can also have an impact on the motivation to run [30]. Studies have shown that middle-class marathoners are conscious to control and regulate their lives through sports [31]. Their motivation to run is also related to their family environment and education level. Sports are more likely to be an option when people have more free and less financial pressure [14].

Secondly, more and more residents are focusing on health issues. Choosing running sports is a healthy choice. Running, a type of endurance training, contributes to cardiovascular endurance and enhances cardio-pulmonary function [10,32]. Fitness-wise, it will increase muscle endurance and prevent osteoporosis [33]. Long-term participation in running can maintain good body shape, lose weight and fat, and increase strength. Moreover, marathons can relieve negative emotions and reduce the risk of depression [34]. The MOMS scale clearly indicates that runners gain a deeper sense of satisfaction and well-being when participating in marathons and therefore they develop a sustained motivation to participate [35].

In the case of running groups in Chinese cities, they represent a place of belonging for sport and emotional attachment. In addition, there are community shared norms of behavior and community culture. With the promotion of running groups, runners naturally embrace the idea of continuous movement. Social transformations have been influencing the transformation of urban life, social interactions, physical activity and sport participation. Nowadays, more and more forms of sports participation also show people’s interaction, interactions and emotional needs [31]. With the support of social interaction theory, this study investigates the sports participation and interaction patterns of running societies, hoping to shed light on the development of urban running societies and urban sports participation.

## 3. Materials and Methods

Instead of focusing on simple group activities, this paper provides an in-depth analysis of the principles of social interaction that exist in running groups and, in turn, focuses on the intrinsic demands and expectations that runners have of running groups. This study used in-depth interviews and participant observation for data collection. The observations and interviews were conducted by two researchers, and one researcher also participated in activities in the urban running group during the study, conducting five participant observations and numerous informal conversations with members of the running group. The researcher took notes on the observation process.

### 3.1. Participants

This study used snowball sampling to recruit volunteers to find groups relevant to the study, with a sampling method reference [36]. The interview outline was determined through pre-research and, based on the need for the study to ensure that respondents could provide a more comprehensive experience of sports participation, we limited the size of the group from which the volunteers were drawn to 300 or more. Five different types of urban running groups were selected in order to gain a more comprehensive understanding of the commonalities and individualities of social interactions in sports among people of different social classes and genders. In this study, we did not consider the age distinction in the selection of the sample, but focused on the participation experience of runners, so we selected participants with more than 3 years of participation experience, who have richer participation experience and can provide more information about the interaction. Secondly, unlike the training of professional athletes, Urban Runners is an interest-related group that participates in the general population in the city. Their activities are all related to running, and runners are required to complete more than 5 km of running per activity, and the average speed is not clearly defined, so there is no restriction on the training standard of participants in this study.

The selection criteria for participants were as follows: 1. located in economically developed provincial capitals in mainland China; 2. participated in running groups of 300 or more people; 3. participated in urban running groups for more than 3 years and had running experience; 4. participated in group running activities at least twice a week; 5. ran 10 km or more per week. In order to find groups with similar experiences, after formulating the initial selection criteria, the research team visited three different types of running groups with more than 300 people in the city and recruited two members of urban community running groups to participate in the study. A snowball approach was used to recruit volunteers until the data were saturated, and the final participants were 10 members of 5 urban running groups, including 5 men and 5 women, aged between 24 and 50 years old (Table 1).

### 3.2. Data Collection

Regarding the determination of the sample size for the qualitative survey, we mainly rely on the Golden Standard of sample size proposed by Walker (2012) and Malterud (2016) [37,38], where the collected information reaches Data Saturation and Theoretical Saturation. During the study we conducted 2 rounds of in-depth interviews, each lasting 45~90 min. Interviews were conducted in public places, such as the activity rooms of running club groups and cafes. Interviews included general and broad open-ended questions about participants’ activities, behaviors, and experiences as a member of a running group, and participants were asked to describe their different running experiences in different social contexts (including school, home, and work). Participants described their experiences of being part of a running group and how they “self-expressed and interacted” in their sport participation.

The core questions:

1. “Why did you join the running group and what is your attitude towards the running group?”.
2. “What are the activities of the running group?”.
3. “how do you relate to other members?”.
4. “what are the rules and requirements of the running group?”.
5. “What are the impressive things/stories from the activities?”.
6. “How have you changed since joining the running group?”.

The researcher conducted continuous data collection from March 2021 to November 2021, and the material from each interview was transcribed prior to data analysis. The three researchers combed the collected data for the vein, similarities and differences between meaning units, categories, and themes, and determined that the data were saturated with richness and thickness until the collected data were repeated and no new themes were found. The three researchers agreed to stop collecting after saturation.

### 3.3. Data Analysis

To protect the identity of the participants, their real names and affiliations were removed, and the anonymity of the members was guaranteed in a specified protocol. Each interview transcript was transcribed verbatim, and audio files were transcribed and sent to participants for member checking to ensure accuracy. The first author processed the recordings and transcriptions with the help of the second author, listening to them and taking notes to identify the most salient themes. After the first author began analyzing the transcriptions, the other two authors read and independently coded the transcribed text. The authors discussed the transcripts with each other, and we identified several themes through a thorough reading of the material, looking for patterns and similarities. After completing the initial coding, the three researchers met to discuss code changes, evaluate generic codes and review new themes. We analyzed interviews and organized findings based on excerpts from conversations with members of the running community to find any additional insights to prevent any missing concepts. The first author also provided the data collected from participant observation to other researchers for further comparison and information review. Our interest was to explore the content of the dialogues and identify their main themes, focusing on the expressions of the association members. The excerpts were selected because they were considered relevant and contributed to our research questions.

## 4. Results

### 4.1. Self-Presentation and External Motivation: An Analysis of the Interaction among Runners

As a matter of fact, in the developing process of marathon running groups, interactions between runners are rather frequent and represent an indispensable part of the group. Different members joining the running group can make the interactions more diversified and richer; they can play the role of “catalyst”. In terms of the interactions of members, it hinges on the common interest, free from any restriction of identities. Interacting out of pure interest and self-presentation is surely more authentic to showcase one’s sports needs and manifest their views. Hence, for participants, he or she will expect responses from participating in marathon activities, where they can show their genuine emotion, constructing self-identity and realizing self-esteem [6]. In running groups, runners present themselves through online or offline interaction, and the interaction is filled with personal expectations of self and others’ expectations of self. Yet such interaction is suffused with personal expectations of self, as well as others’ expectations of self. Additionally, the American sociologist Cooley coined the term of “me in the mirror”, suggesting the three stages of human interaction, namely, my expectation of others’ evaluation, others’ ratings and attitudes toward me, and my imagination of others’ evaluation and the disciplinary role it plays [39]. In the case of marathon enthusiasts, all along, running performance and running technique remain the main topics of communication. Interviewer ZG mentioned as follows: “*The one who has outstanding performance will be role model for the entire running group. And more often than not, people would ask him for advice. As a result, the one with best performance surprisingly exchange more views with the one who has mediocre performance*”.

ZG, who had just finished a 5K run at the time of the interview, said he had been running at least four times a week. At the age of 50, he began to love running passionately and pay close attention to the updates of his fellow runners.

*“Every time I share my running results to my running buddies, I will be encouraged and more motivated”*.

(From HLP’s interview)

In the interview, XY referred that he had been urged to keep running. *“My running group organize weekly running activities, and as of today, it is the 25th session. We have agreed to run over 5 km twice a week. But I was too hectic last week and missed another run. So other group members kindly reminded me of making up for the run this week”*. XY and his fellow runner YDX in the running group often meet to run together, to urge each other, and to remind each other as well. YDX mentioned, “*he and I met in the running group, we have been running together since last year. Whoever is late for running will buy another one coffee*”.

**Figure 1:**
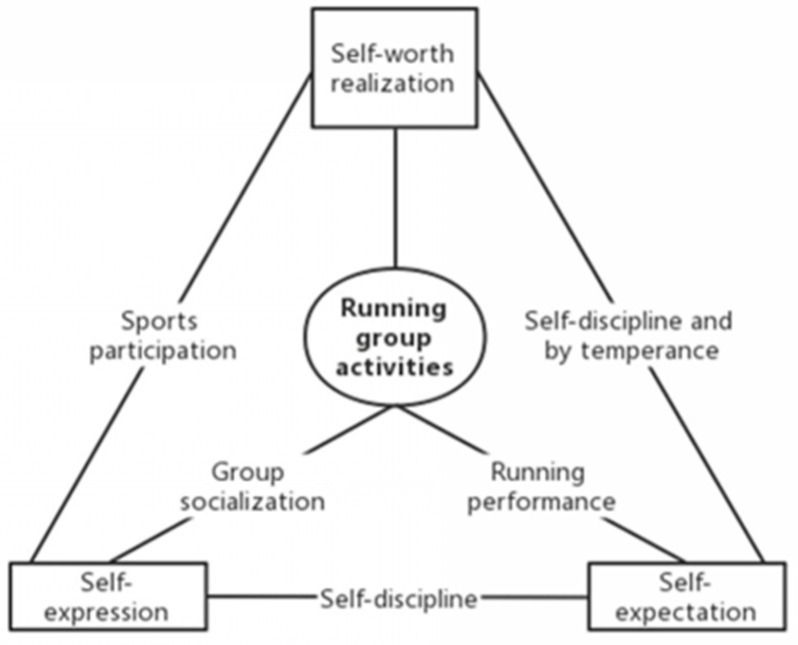
Relationship diagram between runners’ sports participation and interaction.

In running communities, individuals can actually influence and constrain each other, progressively shape their thoughts and responses with certain behaviors under the influence of the running community, while individuals also exert a counter effect to the community, and vice versa (Figure 1).

Regarding group members’ communication, running performance is the main topic of conversation and drawing much attention of members. In more specific terms, most of the interviewers referred the importance of performance during their communication. Those with distinguished results naturally receive more attention. According to Thorstein Veblen, all along, recreational sports have been deemed as a means of proving one’s social status and prestige. Whereas for running, this is not the case, after all, there is no pressure imposed by external status; instead, people will concentrate on running performance and win others’ recognition. Topics, such as the mileage, the average pace, and even the running route of the daily running, will draw other members’ attention and become hot topics. By posting their running results online and making running appointments offline, members of running groups carry out another form of social activity. In fact, they construct external discipline simply by offering “likes” in group chats online and urging each other in the process of running. A runner’s self-expectation and the motivation of running friends, in fact, function as self-discipline and discipline by others, which in turn, reinforces the transformation process of self-behavior after internalizing it [40]. Aside from this factor, the interaction between individuals provides an opportunity for strengthening such discipline so that it can help runners develop sustained running behaviors. The interaction with other runners also amounts to a process of seeking recognition from others. After all, for frequent runners, running has become a part of life; the identity of runners can both satisfy the psychological needs of the self and enhance their social value concurrently.

### 4.2. Self-Demands and Group Discipline: An Analysis of the Interaction between Runners and Running Groups

The individual–group interaction model, as the term suggests, refers to a direct interaction between an individual and a group of people; such interaction aims to blend in a group, the purpose of doing so is rather complicated in most cases, because there is likely to be special needs between interactive process, while the process may last longer with possibly tortuous and variable communication procedures [39]. On the part of group members, they observe common behavioral norms, are emotionally dependent on each other, influence each other in their thinking patterns and even share mutual goals to strive for [41]. As a matter of fact, both the behavioral norms established by the group and its culture serve the interests of group members. While group theory considers the reference group as one in which an individual’s self-recognition would set and maintain standards for the group and provide a framework for comparison [42], group theory also brought forth a group socialization model to describe and explain the mobility of individuals through groups. In the actual participation in marathon running groups, the relationship between the individual and the group determines the development of the group itself; at the same time, runners engaged in group interaction will impact each other and eventually establish and solidify running society culture unique to each running society. Runners participating in the interaction will be bound, consciously or unconsciously, by the values and codes of conduct embraced by the running group culture; then, individuals will internalize the group culture, and actively implement the codes. This is how the culture recognition is projected into the group consciousness and influences other group members. Any group participation and form contain the creation of a new culture and the integration of self-worth consciousness, i.e., the revising of self-worth consciousness to form similar value consciousness [9].

*“Member of the running group who never participate in the activity will be given a heads-up, warned; if posting irrelevant content to the sport in the group chat, he or she will be kicked out from the WeChat group”*.

(From ZG’s interview)

*“Everyone consciously abides by the rules of our running group, such as not to send advertisement links, and not to have others join in without the consent of the WeChat group leader”*.

(From LI’s interview)

*“In fact, the majority of members participate in activities quite often, and there are few compulsory measures for those who do not participate in any. But we do network little with such members”*.

(From HLP’s interview)

The first interviewer, ZG, along with the sixth interviewer, LI, and the fourth interviewer, HLP, all mentioned the basic norms in their running groups, but there are no mandatory measures; after all, running groups fundamentally follow individuals’ wishes and merely require members to observe the most basic codes of conduct. When asked if there were any cases of quitting the running group, ZG answered like this:

*“The main reason is that there are already way too many WeChat groups on my phone, and the message keeps annoyingly popping up all the time, So I quit many groups. At the time being, I just participate in one running group and quit all others. Honestly, I used to join in similar groups, but I had never engaged in any activities, I was somewhat embarrassed to be in it”.*

There is no strict entry and exit mechanism for running groups, and runners can choose to join in or quit the group according to their own wishes. In fact, at the time the phenomenon of “social network overload” occurs, people will consequently reserve their time and energy, but allocate them to the groups they are genuinely interested in [43]. This is how runners screen out and choose running groups, quitting those that are less appealing to them or just keeping being in the group in a stealthy or “invisible” manner for a long run. Runners of this kind will inevitably feel like “others” and not fit in at all [44]. Consequently, they might lose the opportunity to further interact with information publishers for one thing, and they may be forgotten and marginalized by other members for another, because they do not engage in any interaction, such as chatting in the WeChat group. When they are unable to maintain effective interaction with the group, they are quite likely to terminate the status quo and look for other running groups.

*“We often play solitaire in the WeChat group, sharing the mileage we have covered in a weak and the total mileage in the last six months. And then we will discuss on this topic”*.

(From ZH’s interview)

*“It’s my routine to save the share the record made by the Joyrun, an app advocating the new running way of life, and everyone in my WeChat group would give my posts “likes”*.

(From XY’s interview)

*“I usually share my running record in the group, not for showing off how fast I ran today, but for proving I keep insisting on running. That’s my reason of sharing, it is also a way of encouraging myself. The more I share, the keener interest I would have in running. By doing so, I do construct my own sense of identity towards running, this positive hobby”*.

(From ZG’s interview)

**Figure 2:**
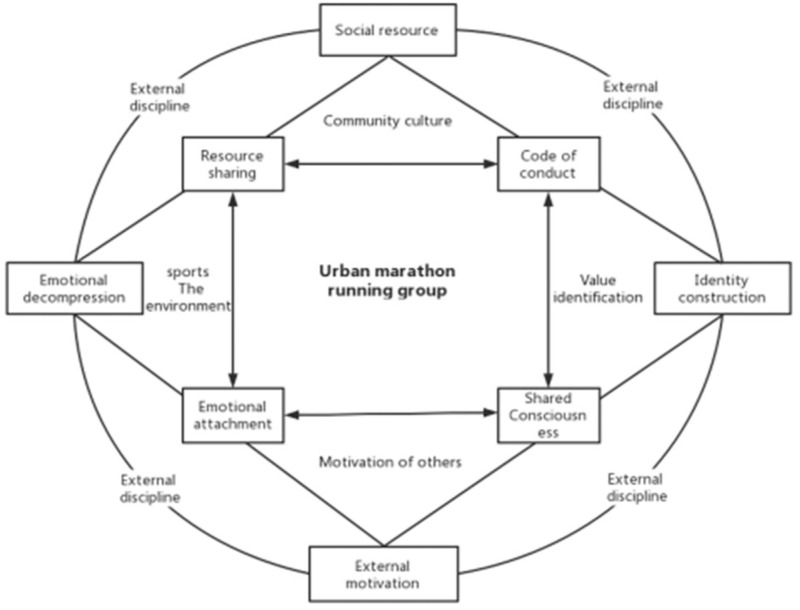
Interaction relationship within the marathon running group.

ZH, XY and ZG all have shared their running records in running groups. Through mutual communication and encouragement, their bond with other members, and the intimacy between groups can be enhanced in no small degrees. Newly joined members can also establish a sense of identification with the group culture and group consciousness through interaction. According to Tajfel, when an individual mentally deems himself or herself as belonging to a certain group, the identity of group membership will entail a sense of emotional support and merit for the individual [45]. Members of running groups create and maintain a relaxed and enjoyable vibe during their interactions, free from any psychological pressure, burden, or bearing excessive emotional costs. In fact, members of marathon running groups are highly focused on running performance and initiate the interactive activities self-consciously between themselves and others. When the information they post obtains a positive response, runners will feel the satisfaction of being recognized and living up to their inner expectations, which are all derived from the WeChat group. Therefore, their interest in running will be solidified in turn. It is right that, with constant mutual encouragement, runners are more spurred to identify with themselves, the running group, as well as the members. Such practice facilitates the construct of shared consciousness, injecting it into the running group culture (Figure 2).

*“After joining the running group, we all run together, and there is a significant improvement in our morale, which also exert positive impact on our lifestyles, such as few of us would stay up late. For me, back in my college days, I always burned the night oil, even for no good reason. Yet after running, I do become more self-disciplined”*.

(From ZG’s interview)

*“In the running group, the vibe of keeps practicing together is quite intense. Otherwise, one may lose interest in this activity soon providing that he or she just a beginner, or just did not see any achievement in a short term. However, if there is a group with various activities that he can join in. The more he takes part in it, the more interest he will have, and the better such a habit can be developed”*.

(From YDX’s interview)

The theoretical knowledge and running techniques offered by running groups can make runners blend better into them and develop good habits, which in turn helps young runners achieve individuation in society. The social identity that runners have will be more prominent when they are in running groups, and it provides a way for runners to transcend themselves. Runners establish behavioral and psychological connections with the group, actively accepting the group culture and group identity, and hence, the interactive chain between the self and the group can gradually come into being. When they are subject to the long-term influence of group culture, runners will be motivated and urged to shape a continuous wish to run. The running activities, especially when it comes to marathon running, bear a deeper connotation, and they will naturally become ways to express oneself, and evolve into a form of loyalty. While for runners, they can achieve self-improvement and progress via interaction with group members.

### 4.3. Intergroup Interaction and Group Identity: An Analysis of Intergroup Interaction in Running Societies

The occurrence of group interaction is a gradual process, which gradually develops from individual to individual to one-to-many and many-to-many interactions in three dimensions [46]. When the level of interaction gradually increases, the objects it contains will increase, the topics will be richer, the communication time will be longer, and the interaction process will be complicated. There are differences in the size, organizational operation, and management mode of running community groups, and it is precisely their differences that make the dynamic changes between running communities and running communities exist [47]. The relationship is characterized by competitiveness as well as negative reciprocal attitudes. The connection between running societies always unfolds in the competition between each other, and runners, through the process of screening and selection of running societies, will deepen their identification with the running society they belong to, showing preference for the in-group and prejudice against the out-group. The achievement and positive influence of the inter-group must be obtained and derived through the isolation and negative influence of the outer group [48]. As a result, the identity of the running group to which runners explicitly belong influences their attitudes and behaviors and entails recognition and positive evaluation of the culture of the running group.

*“My running group is a branch of YPT’s running group. Every activity the group holds have explicit principles, purposes and slogans with staff bearing a clear division of labor. Every month my running group will provide free drinks and fruit, and occasionally there will be small gifts, such as mugs, stickers as souvenir etc”*.

(From YDX’s interview)

*“Honestly, I have tried many running groups, but RPT running group is the one I’ve been in constantly. Because the atmosphere of the whole running group is very good, and in the last activity, I was given a towel exclusive to our group. It was printed with a logo, which is quite practical and good-looking as well”*.

(From ZH’s interview)

According to YDX and ZH, different running groups have their own iconic slogans, philosophy, and costumes. As the meaning carrier, or symbols, such propaganda tools represent the unique group culture of its own. In terms of the activities of running groups, symbols play an important mediating role in the process of people’s social interaction, which will implicitly influence runners and enhance their members’ identification with the group culture [39]. The meanings of these special symbols are apparently created by runners consciously, and also constructed in the process of mutual interaction; with such endeavor, consciously and unconsciously, the symbols that running group members agree on come into being [30].

In ZG and XY’s running group, the activities are organized in a more casual way compared to YDX and ZH’s. It has neither redundant group regulations nor notable symbols.

*“The running groups I created are of small scale, serving teachers and students mostly. The activity is quite spontaneous and casual. Whoever feel interested in can join in on voluntary basis. And basically, they run at their own speed, lasting for 30 to 40 min. There are 159 people in the running group, about 10 are frequent participants”*.

(From ZG’s interview)

*“The running group I am in is a relatively big one that runs around hills. Together group members go hiking on weekends. Running group of this nature usually have more diversified activities, each time it will design some routes in advance, which is quite interesting. So, each time there are some 40 runners participating in activities”*.

(From XY’s interview)

Without any doubt, different running groups have different characteristics in terms of their management mode, organization of activities, and the running group culture. The appealing degree to members of running groups also varies in no small degree, so is the case with the number of daily active members. Generally, the richer the activities of a running group and the more standardized the organization is, the more constant and active members it will attracted.

*“I’ve been in the running group for quite a long time. Two years ago, the running group was not so well developed, which was merely led by a leader, and people who joined in were simply out of their interest. Now, we have a group leader, a deputy leader, and an administrator. And we usually summarize the pros and cons of the activity after it ends, and also bond with other running groups. So, it is getting better and better”*.

(From LI’s interview)

*“We often forward some marathon information in the group chat, because I am in a few running groups, so the time I see any useful information I will share with the group members”*.

(From ZH’s interview)

**Figure 3:**
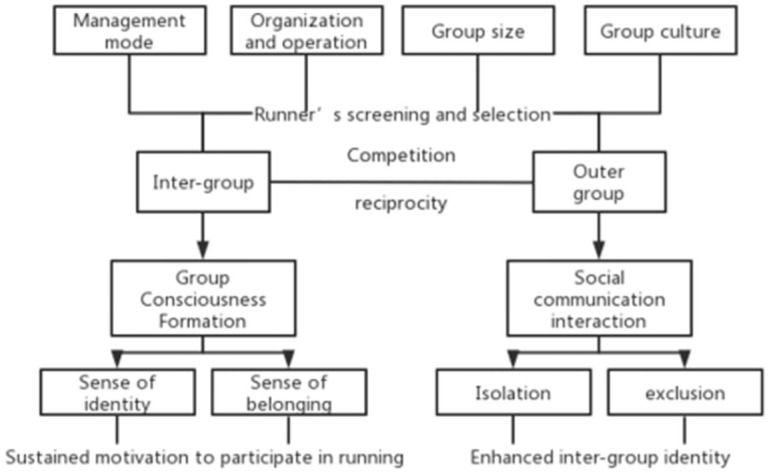
Interaction between marathon running groups.

As to the marathon competition, there is also a certain interaction between different running groups, because each individual is the embodiment of the group culture. In this context, a quality running group can have a large arena to manifest its running culture, appeal for more members to join in so as to promote its development, especially its management norms, organization activities, establishment of group consciousness, and the enhancement of the sense of identity and belonging of members. What merits attention is that, at the time the individual participant actively distinguishes themselves from other groups, his or her perception of the group will be strengthened. As a matter of fact, via the four stages of information origin, information diffusion, social interaction and cohesion formation [49], the gradual evolution process of the gestation and development of group consciousness is progressively completed. Nurturing the group member in this way, the running group can therefore cultivate members’ consciousnesses, making them more interactive within the group and support the group they belong to with actual deeds, etc. (Figure 3).

## 5. Discussion

In study, we found that the interaction of runners is influenced by social transformation on the one hand and the increase in health awareness on the other [6]. People are more concerned about their own health and pursue physical and mental pleasure. The emergence of group organizations, such as running groups, also shows the pursuit of sports and health and the change in people’s thinking.

**Figure 4:**
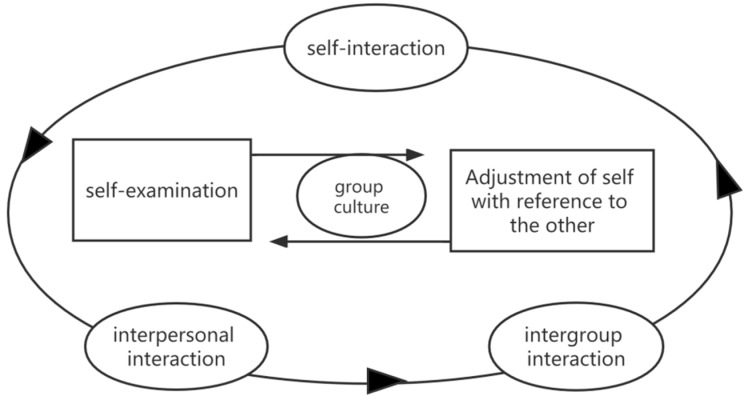
Interactive relationship map.

The running community has also become a place where runners attend for emotional support. There is extensive and close social interaction activity within the group. In the study, we found that many running group members focus on their own progress. They value their self-efficacy more and will gradually find their sense of self-worth in running. They also want more recognition from others, and many participants said self-improvement can bring greater attention, and such attention can satisfy inner expectations. Escandell-Vidal (2016) also mentioned that in social situations “expectations” are more stable and adjust themselves to the environment [50]. We also found that “expectation” is a constraint in the interactions of running associations, and in the social environment, expectation can be transformed in many ways, which can guide people’s understanding and behavior. Runners will constantly examine themselves, discipline themselves, adjust themselves, and correct themselves in the interaction to achieve self-interaction and interpersonal interaction. “Self-examination-reference adjustment” is a cyclical process, in which the two are created in interaction and adjusted in mutual interaction. The group is at the center of the interaction from the beginning to the end of the process, acting in the whole process of interaction (Figure 4).

Yongbao Z (2012) proposed that there are actors, goals, vehicles, norms, and environments in the running group that can have an impact on runner participation [5]. In the present study we found that this interaction is not one-way but two-way. The internal norms of running groups discipline runners and also produce motivational effects. The inclusion of runners in a running group can facilitate the phenomenon of interaction. Interaction in turn allows runners to create a sustained intention to exercise. Whether collaborating or competing, these interactions bring them closer together. This is in line with the concept of Interaction Ritual Chains proposed by Randall Collins (1986). Different types of “encounter” also generate different emotions, which are driven by situational energy and create symbols and signs that represent different groups. Therefore, with multiple interactions, emotional relationships are created and continue to function. This is the necessary factor needed for the creation and spread of the running group culture.

There are still some limitations in this study. In the study, it was found that members in the running group would gradually develop group consciousness and present a preference for in-groups. The attitudes of running group members toward in-groups are less addressed in the current study, and although related studies are mentioned in this study, the relevant content still needs further exploration. Second, as society evolves, age differences also have a great impact on interaction style, quality, and frequency of interactions [51]. This study focused more on runners’ experiences and participation in running group activities in the sample selection and did not further investigate the differences in the interaction situation of each age group; this issue is hoped to be further explored in future studies.

## 6. Conclusions

Interaction in running groups is a process consisting of three stages, i.e., self-interaction, interpersonal interaction and intergroup interaction. The runner completes the whole process of interaction though three aspects, namely self-examination, adjustment with reference to other, and belonging to the group as the core of consciousness.

With respect to the self-examination, it is manifested by the runner’s internal consciousness in the interaction process. The role each person plays constitutes the foundation of a social group; while for each individual, he or she is prone to participate in social interaction, and even impose discipline onto himself according to the norms of the role. The process of role-playing in the running group is also equivalent to the process of continuous improvement of self-knowledge. On the running field, runners constantly push their limits and find their intrinsic merit, while off the field, they scrutinize themselves internally and realize the process of interacting with themselves.

Adjustment of self with reference to the other. Runners interact socially with group members who share the same group identity and features. Ensuring obtaining the recognition of other members, they gradually form a certain discipline of self-behavior and attitude, and constantly adjust themselves. Concurrently, through frequent interactions within the running group, runners’ trust in the group can therefore be enhanced. Moreover, sharing running news, running performance, and running information in the group chat can draw a wide range of responses, and runners break up the social barrier through their giving of “likes”, mutual encouragement and communication, so that the group members’ sense of belonging and identity are solidified.

The group culture is constantly constructed through the interaction of group members. The process of choosing different running groups is equivalent to the process of embracing group culture. The joining of new members and the leaving of old members change the inner structure of the group, and the flow of core members also brings about the change of group culture. Regarding the specific running group, the unique symbolic culture, standardized group organization and harmonious atmosphere of the group can stimulate runners’ interests in running, enhance group communication and establish stable intergroup relationships, and then yield continuous interactive behavior.

## Figures and Tables

**Table 1:** Basic information of interviewees.

No.	Interviewee Code	Gender	Age	Education Background	Occupation	Joining Time
1	ZG	Male	50	Bachelor	College lecturer	2014
2	YDX	Female	26	Master	Public servant	2019
3	ZH	Male	23	Bachelor	Student	2018
4	HLP	Female	28	Master	Middle school teacher	2018
5	XY	Male	29	Bachelor	Freelancer	2017
6	LJ	Female	32	Master	Public servant	2015
7	HXX	Female	46	Doctor	College lecturer	2017
8	LBF	Male	38	Bachelor	Freelancer	2015
9	LY	Male	40	Bachelor	Freelancer	2018
10	LCJ	Female	22	Bachelor	Student	2019

## Data Availability

Not applicable.
